# Child health in Syria: recognising the lasting effects of warfare on health

**DOI:** 10.1186/s13031-015-0061-6

**Published:** 2015-11-03

**Authors:** Delan Devakumar, Marion Birch, Leonard S. Rubenstein, David Osrin, Egbert Sondorp, Jonathan C. K. Wells

**Affiliations:** Institute for Global Health, University College London, 30 Guilford St, London, WC1N 1EH UK; Center for Public Health and Human Rights, Johns Hopkins Bloomberg School of Public Health, Baltimore, MD 21205 USA; Royal Tropical Institute, Mauritskade 63, 1092AD Amsterdam, Netherlands; Childhood Nutrition Research Centre, Institute of Child Health, University College London, London, WC1N 1EH UK

**Keywords:** War, Conflict, Children, Health, Syria

## Abstract

The war in Syria, now in its fourth year, is one of the bloodiest in recent times. The legacy of war includes damage to the health of children that can last for decades and affect future generations. In this article we discuss the effects of the war on Syria’s children, highlighting the less documented longer-term effects. In addition to their present suffering, these children, and their own children, are likely to face further challenges as a result of the current conflict. This is essential to understand both for effective interventions and for ethical reasons.

## Introduction

Civilian populations are increasingly exposed to contemporary conflicts. That children are amongst the worst affected by war is widely known, but the full extent of their suffering is still not clearly understood. This article discusses the effects of the war in Syria on the health of children, with a focus on the less documented longer-term health effects.

The Syrian war is one of the bloodiest in recent times, with no end in sight and has been described by the United Nations High Commissioner for Refugees as the “worst humanitarian crisis of our time” [1]. It began in January 2011 as a civil uprising on the back of the ‘Arab Spring’ movements throughout the Middle-East and North Africa. The government responded to pro-democracy demonstrators with violence, a flashpoint which ultimately led to armed opposition. A civil war between the Syrian government and opposition groups, each with their own supporters, ensued. The conflict has since evolved into a larger, more complex war, merging with other regional conflicts involving the Islamic State and multiple factions across several countries.

## Review

### Immediate effects on child health

Numerous violations of child rights have been reported by the United Nations in Syria. Data from August 2013 showed that approximately 11,500 children had been killed, with “exponential increases in killing and maiming” over the previous year [2, 3]. By May 2015 it was estimated that 5.6 million children were in need of assistance [4]. As of August 2015, 7.6 million Syrians (approximately half of whom were children) were internally displaced and a further 2.1 million children were refugees  in nearby countries [4–6].

In addition to death and displacement, the immediate costs of war are numerous and include injuries, increases in food insecurity (potentially leading to malnutrition) and communicable diseases in poorly equipped and crowded camps for internally displaced persons and refugees. An assessment from 2013 highlighted the level of food insecurity, [7] but data quantifying the prevalence of malnutrition are generally lacking. Some studies have assessed nutritional status in refugee camps. A survey from a camp in Jordan showed a higher prevalence of anaemia in the occupants than in the host population (48 % (95 % CI 42, 55 %) compared to 26 % (95 % CI 21, 31 %)), although the rates of wasting were no different [8]. In refugee camps in Lebanon, an increase in global acute malnutrition (GAM) was shown between 2012 and 2013 from 4 % (95 % CI 3, 7 %) to 6 % (95 % CI 5, 7 %) in children aged 6–29 months; although this was a non-significant increase, under the World Health Organization (WHO) classification of GAM, the nutritional status of the Syrian camp population is considered “poor” (GAM between 5 and 10 %) [9].

Children are affected through direct attacks (sometimes even deliberate homicide or execution), as victims of “collateral” damage (for example, indiscriminate use of aerial barrel bombs in densely populated cities such as Aleppo), and as a result of the systematic breakdown of societal structures [10]. Approximately 1,000,000 Syrian children are currently living under siege or in areas hard to reach due to violence [6]. Whereas Syria’s public health indicators were improving before the war, and the country was experiencing an increase in life expectancy and changing disease patterns from communicable to non-communicable diseases, its health system has now collapsed [11, 12]. In 2014 the WHO reported that nearly three-quarters of hospitals and one-third of primary health care facilities were unable to function, and that hospitals (and also schools) were being used as military bases, exposing them to opposition attack [10, 13]. Water supplies have been targeted deliberately; those of Aleppo, for example, failed after the Al-Khafsah pumping station was attacked and sewage is no longer treated [6, 14]. Increased prevalence of vectors and pathogens, lack of a surveillance system, preventative programs and infrastructure, and likely impaired levels of immunity (as a presumptive consequence of malnutrition and reduced immunization rates, and possibly of stress) have led to a greater overall burden of disease - including vaccine-preventable diseases - illustrated by the reemergence of polio and outbreaks of measles [6, 15].

### Long-term health effects

The above rightly focuses attention on the immediate plight of Syria’s children, but evidence increasingly suggests that the stresses of war can have less visible effects that last for years or decades. Children who survive trauma may be left with lasting disability and mental scars, with consequences for their future health and social and economic life skills [16, 17]. Rates of trauma can remain high after conflict, and longer-term psychological and psychosocial effects may be aggravated by a combination of the increased presence of weapons and normalization of violence within society [18]. Acute exposure to violence can lead to mental illness, such as post-traumatic stress disorder (PTSD) and anxiety, which can persist well beyond the conflict [19, 20]. A systematic review of mental health in refugees and displaced people in Syria and surrounding countries (including 13 studies) found high and rising levels of mental distress but also highlighted methodological difficulties in obtaining accurate prevalence figures for mental illness. In children the symptoms included nightmares, bedwetting and changes of behaviour (aggressiveness or being withdrawn). One study of children in Lebanon for example, showed an unusually high prevalence of PTSD of 76 % [21].

In 2013, 2,000,000 children were estimated to be undernourished in both macro- and micronutrients in Syria, [22] which in early life alters growth trajectories, propagating effects over the life-course and affecting adult stature, risk of illness and potential earning capacity [23].

Societal changes can also be long-lasting. Breakdown of community structure results in children taking on roles reserved for adults at the expense of education and loss of future earnings, [24–26] an extreme example being their use as child soldiers [27, 28]. War can provoke family breakdown through death and displacement, and can also change the roles of remaining family members [29]. Half of school-age children within Syria and two-thirds of Syrian refugee children are not in school [30]. It is estimated that this will cost the country up to 5.4 % of its Gross Domestic Product if in the long term the 2.8 children this represents never return to school [31]. This is compounded by an exodus of the educated population that is likely to delay post-conflict recovery [10].

### Inter-generational health effects

In addition, the effects of conflict are likely to be felt by children yet to be born. As previously described, war is a pervasive environment in which trauma, infectious disease, mental illness, and poor nutrition can affect maternal physiology sufficiently to propagate biological effects across generations. We discuss the evidence for this in a related article on this topic [32].

Based on information from other conflicts, increases in rates of preterm birth, fetal growth restriction, and maternal infections leading to congenital abnormalities are highly likely to increase [32]. Data from the Syrian conflict are currently sparse, but a study of 452 Syrian refugee women in Lebanon highlighted some of the problems. It found barriers to antenatal care, common exposure to violence (31 %) and a high rate of preterm births (24 %) [33]. Rates of Caesarean sections, with their associated morbidity, were also high (45 % of deliveries) as women were afraid of giving birth at unpredictable times in insecure environments [15]. There is similar evidence from Syrian refugees in Lebanon, where rates of Caesarian sections were 35 % (of 6366 deliveries assessed) compared to approximately 15 % previously recorded in Syria and Lebanon [34, 35]. Though relatively small studies, similar outcomes would be expected in the nearly 40,000 babies already born as Syrian refugees, where coverage of adequate antenatal care and skilled healthcare workers at the time of birth is lacking [6, 36]. The mechanisms by which intergenerational adverse effects occur are complex. Increases in maternal trauma (including rape and intimate-partner violence), infections, lack of medication, illicit drug use, poor diets and stressful experiences all play a role and are all seen during conflict [32, 37].

Future Syrian children may be affected by the current conflict through inadequate nutrition. Reports from Syria exist of reductions in breastfeeding and the increased use of breastmilk substitutes [37]. This can be as a result of maternal stress and malnutrition that leads to inadequate nutrition and an impaired immune status for the infant. Increases in food insecurity after reduced crop production, breaks in the supply chain, and the breakdown of the economy can lead to lasting changes in food supply [22]. Sieges are an extreme example, imposing mass starvation. Examples include the siege of Homs, a 3-year battle between the Syrian military and opposition forces during which a lack of food led to reports of people being forced to eat grass and weeds, [38, 39] and amongst Palestinian refugees in the Yarmouk refugee camp [40]. Studies of the Dutch Hunger famine (World War II) and the Biafran conflict (1968–70) have shown that maternal famine can increase the risk of chronic diseases such as diabetes, hypertension and cardiovascular disease in adult offspring [32]. Increases in maternal stress and mental illness, common in war even among those not directly exposed to violence, are associated with changes to the child’s hypothalamic-pituitary-adrenal system (via epigenetic changes to glucocorticoid genes), leading to an increased susceptibility to mental illness [41]. Changes to germline cells may also propagate transgenerational effects to the grandchildren of those affected [42]. In long-lasting conflicts, a combination of direct exposure to acute trauma and a reduced capacity to cope with stress due to intergenerational effects may lead to an exacerbation of symptoms [32].

## Conclusions

Warfare affects both today’s children and those yet to be born in ways that can last a lifetime. The war in Syria may seem an extreme example, but has much in common with previous and other ongoing wars. While our understanding of the longer-term and inter-generational effects remains limited, these issues are important for many reasons, including a need to project future public health burdens and their implications for long-term support, as well as for their ethical implications. An appreciation of the full health and inter-generational consequences of war also has implications if parties to the conflict are to be held accountable under international human rights law, including the right to the highest attainable standard of health.

At this point in the war in Syria, at which there appears to be increasing fighting, we need to focus on the needs of vulnerable populations (including those yet to be born) who have already suffered and continue to do so and, in addition to providing immediate humanitarian aid, provide a coherent strategy for the future. An example would be ensuring adequate coverage of the ‘Minimum Initial Services Package for Reproductive Health’ for women and girls, which has the potential to reduce the harm caused both to them and their children [43].

While much work has been done to improve the accuracy of data collection during conflict in recent decades, the challenges of obtaining accurate data are still acknowledged [44–46]. These include a lack of security, rapid population movements, breakdown in health information and surveillance systems, and the manipulation of health information by parties to the conflict. However the information that does exist about the effects of war on child health in Syria, and the information that is known about child health in conflict more generally, makes a strong case for this to be a consideration in the planning of services post-conflict. It is also a clear indication that parties to conflict urgently need to take the full impact of conflict on children into account in their actions. The existing information presented here is a basis for further research to strengthen these arguments and we would encourage the collection of systematic data on health from the early stages in a conflict to enable a more appropriate humanitarian response and longer-term planning.

The tragic harms experienced by Syrian children in the current conflict are only some of the difficulties that they and their future siblings and children may face. Children are undoubtedly resilient to multiple stresses, but this capacity is limited and every effort must be made to mitigate harm where possible. An end to war must be given top priority by the international community and all parties within the conflict. In addition, the children who have suffered so much from the conflict in Syria must be followed and health data collected, both to understand the intermediate and long-term effects of the conflict, and to ensure that interventions are designed to ameliorate the harms that have befallen them.

## Abbreviations

PTSD: Post-traumatic stress disorder
GAM: Global acute malnutrition
WHO: World Health Organization
